# Funds and Fair-Play for the Voluntary Hospitals of London

**Published:** 1892-01-02

**Authors:** 


### FUNDS AND FAIRPLAY FOR THE VOLUNTARY HOSPITALS OF LONDON.

OUR appeal to the Metropolitan Press for support in the endeavour to arouse public attention to the importance of giving more money to our voluntary hospitals has produced good results. One or two of our contemporaries have taken occasion to talk of hospital scandals, but when those scandals are examined, they will be found to affect mainly, if not entirely, the rate-supported and endowed hospitals which do not appeal to the public for money. It is most desirable that every member of the public shonld clearly understand that there are three great groups of metropolitan hospitals, (*a*) the endowed, (*b*) the rate-supported, and (*c*) the voluntary hospitals to which last class alone the public are asked to give their money. They may do this with every confidence that that money will be well spent, and that the great voluntary hospitals of London are, on the whole, the best administered and most efficient in the world. This fact was testified to by Lord Sandhurst in a recent speech at the Mansion House, and he, if anybody, certainly ought to know, seeing that he is the chairman of the Lords' Committee, and has taken the closest interest in the evidence produced before that body. Lord Sandhurst has besides made a tour of continental hospitals, and, as a result of his inspections and investigations. he has become convinced of the excellence of the management of the voluntary hospitals of this country. We have dealt with the matter fully elsewhere, but we desire here to impress upon everybody who can afford to give even a little money to charity, that whatever reason he may have for not giving to hospitals, he cannot in justice withold his contribution on the ground that his money if sent to the hospitals will be misapplied or extravagantly squandered. Taking the administration of the voluntary hospitals as a whole, it may be fairly described as excellent. Indeed, it is remarkable, having regard to the volume of work which has to be done, and to the enormous number of persons engaged in the work, that so few mistakes should be made, and that on the whole no just person can apply the word scandal to anything which has occurred in connection with the voluntary hospitals of London during the year 1891.

**Daily News of Dec. 19th, 1891.**

Our contemporary THE HOSPITAL, publishes this week a Christmas number and supplement, but the note of cheerful optimism appropriate to the season is sadly missing in its pages. Our hospitals are still in a bad way, and unhappily their prospects are not improving. It is anticipated that the deficit on the operations of the present year instead of being £84,000 as in 1890, will be not less than £150,000. The writer draws a vivid picture of the extent of the work done by our hospitals in the way of relieving human suffering. Among the most astonishing of its details is the fact that there are computed to be between 250,000 and 300,000 accident cases treated every year by the metropolitan and suburban hospitals alone. Last year Guy's treated the enormous total of 20,766. Of these, 1,297 were accidents of a serious nature, such as broken legs, fractured thighs, smashed ribs, fractured skulls, dangerous injuries from falls or from falling buildings; indeed every conceivable variety of hurt which can come to an industrial population that carries on its daily work in the hurrying streets of the largest and most crowded city in the world. Well may this writer bid us ask ourselves where London would be without its hospitals.

**Daily Telegraph for December 26th, 1891.**

Mr. H. C. Burdett, whose work in connection with hospitals is well known, asks us to remind the public, at this appropriate time, of the needs of those beneficent institutions. Owing to depression of trade and widespread shrinkage of income, it is calculated that the authorities will have to face a balance against them of £150,000. This is a startling deficiency to be raised by voluntary contributions. Some persons have suggested that it should be met by a rate, but Mr. Burdett, after a quarter of a century's experience, is convinced of the superiority of the voluntary over the rate-supported institutions.

**The Morning Post for December 26th, 1891.**

There can be no institutions more deserving of support than the medical charities of London. Their present efficiency was, as our correspondent reminds us, justly asserted a few days ago at the Mansion House by Lord Sandhurst. Lord Sandhurst, who spoke with authority, not only as having presided over the sittings of the Committee of the House of Lords, but also from personal experience at home and abroad, maintained that, with one notable exception, there is no hospital in Paris, Vienna, or Berlin which is equal to the hospitals of London. It cannot, therefore, be contended that our metropolitan hospitals have been careless or unprofitable stewards of the sums which have been placed at their disposal. They can fairly ask to be supplied with the necessary resources to enable them to perform their beneficent functions as efficiently in the future as they have done in the past, and there is no possible excuse for any falling off in the contributions by reason of inefficiency in the work of these hospitals considered as a whole. Funds, however, are badly wanted in all directions, not only to meet the increased demand for hospital accommodation and relief which the ceaseless growth of the population of the metropolis creates, but even to maintain the work at the level which has already been reached. . . . .

Now, the future of a public institution which is compelled to live upon its capital is not less fraught with danger than that of the private individual in the like predicament. Things must inevitably go from bad to worse unless the necessity which compels the realisation of capital be removed either by the generosity of the public, as it ought to be, or by the diminution of the relief afforded to the metropolitan sick poor, as it certainly ought not to be. What the effect of any serious curtailment of the extent of the work done by our medical charities would mean can only be estimated in the light of some knowledge of that work. Only a few days since we called attention incidentally to the fact that the number of accident cases alone which are treated in the course of the twelve months are estimated at more than a quarter of a million in number. . . . .

It is nothing less than shameful that recognised authorities on this subject should be compelled to estimate the total deficit in the incomes of our voluntary hospitals for the present year at the enormous sum of £150,000. Englishmen have always boasted, and, on the whole, with good reason, that the free play of individual enterprise, which is peculiarly congenial to the spirit of our nation, is productive of better results than any plans of highly centralised administration or State support. Our hospitals, in particular, have constantly been quoted as striking examples of the truth of that doctrine. Voluntary support has made them what they are, and that particularly English method will, we are convinced, maintain them at their present high level of excellence better than any cut-and-dried invention which latter day County Councillors can possibly devise. But, clearly, the time has come when the voluntary system in this matter must exert its own enormous latent capacities with considerably greater vigour than it has done of late. The opponents of the system of the voluntary support of medical charities must not be allowed the opportunity to point to such deficits as that which appears at the close of 1891, and which is credibly reported as likely to be still larger in the future. . . .

The inevitable conclusion from all the facts is that the stream of benevolence has been diverted from those channels which most require it, We can only hope that the next twelve months may witness a return to the fundamental doctrine of common sense in the distribution of all charity, namely, that the greater the need of the applicant the greater is the obligation to give prompt and generous relief. If that principle were always put into practice the voluntary hospitals of London would never want for money.

**Daily Chronicle for December 21st, 1891.**

In the midst of comfort and preparations for feasting, to be reminded that the claims of the poor and ailing press for attention, should evoke sympathy of the most practical kind. The hospitals of London, have, it appears, not been adequately supported throughout the year now hastening towards its close. In the current number of The Hospital we find it stated that there is a probability that there will be a deficiency of income for the year as compared with the expenditure, which will amount to £150,000. It is well known that it has required the most strenuous efforts for some years past to maintain in an efficient state the institutions for the sick and needy, which alleviate so much human suffering and save so many lives; but the drain on the resources of these institutions is far in excess, as a rule, of the supply. We learn that, vast as is the noble work performed, the amount disbursed by the executives of hospitals in the year does not exceed £606,258. It will probably surprise not a few people to learn that little more than half of this sum is provided by subscriptions. The population of London, in its most extended sense, is in round numbers 5,657,000, and the sums subscribed throughout this area last year made a total of only £320,662, which would represent but a very small sum per head. The invested moneys of these institutions yielded but £151,229; it will, therefore, be seen that unless the public are prepared to allow much of the benevolent work hitherto done to be abandoned, and whole wards to be closed, more funds must be provided, and there certainly could not be a more appropriate time to remind people who have the means to give liberal help than at the approach of Christmastide. There are some interesting figures which might be cited in proof of the extent of the humane work done in the hospitals. It is estimated, for instance, that there are between 250,000 and 300,000 injuries caused by accidents treated in the course of twelve months. Of these, it may be mentioned, that nearly 21,000 have been treated at Guy's Hospital alone. Rather more than eighty per cent. of all the "accident cases," we learn, were men and women dependent on their labours for their living, and of course the great mass of these unfortunate people would by their disablement be incapacitated for a time from providing for those persons they were in the habit of supporting. It is not for us to suggest in detail how the money needed to carry on the work should be raised. An organised effort is evidently required beyond those usually made, and this time of peace and goodwill towards men is peculiarly appropriate for commencing such an effort.

**The Daily Graphic** for December 25th, 1891.

The picture of "London without Hospitals" which has just been drawn by The Hospital, cannot, with the figures of receipts and expenditure before us, be regarded as needlessly alarmist. A prospective deficit of £150,000 is an item which speaks for itself. Depending as so many of the hospitals do upon public support, it is certain that any large withdrawal of that support must lead, first to decreased efficiency, and ultimately to closed doors. In more than one case which will occur to the minds of many people, it is no secret whatever that the pecuniary resources available hardly warrant institutions of national importance in keeping open. This is an evil which can and should be remedied forthwith. We need say nothing of the work which the London hospitals do day by day. Not a tithe of their usefulness is made known, but broadly the charitable public are fully aware of the immense usefulness they exercise. It might, perhaps, silence their detractors if their labours were performed a little more fully under the public eye. It would, of course, be needless to give gruesome details or trespass upon personal privacy, but in these days of fierce competition for the support of the benevolent, a great deal of unnecessary, if natural reticence is observed. Be this as it may, it is assuredly the duty of the public to see that no commercial depression and no new fashions in philanthropy restrict the usefulness of our great hospitals.

**The Globe** for December 26th, 1891.

It is very depressing, at the close of a year which has been fairly propitious to British trade, to learn that the London hospitals have, speaking generally, fallen into a more embarrassed condition than ever. The total revenue of these good Samaritan institutions during the present year amounts to £522,085. But the expenditure has run away with £606,258, and although the Saturday and Sunday collections wiped off rather more than half of the deficit, the invested capital had to be depleted to the extent of more than £30,000. The regular income is derived from three sources: Investments yield £151,229; public benevolence subscribes £320,662; and patients' payments bring in a trifle over £50,000. It will be seen at a glance, therefore, what a very serious matter it is to diminish the invested capital by over £30,000. But a writer gives solemn warning in The Hospital that next year's deficit will be very much larger. He has "unquestionable authority" for estimating it at "not less than £150,000. Assuming, then, that the Saturday and Sunday collections amount to £50,000, no less than £100,000 will have to be obtained by selling out capital, thereby diminishing in perpetuity the future income from that source by £3,000 or £4,000. Only two alternatives present themselves; either the public must largely increase their subscriptions, or the hospital authorities may feel compelled to economise at the expense of efficiency by closing a considerable number of wards. But this latter course would inflict such terrible suffering on the sick poor that the Government would be bound to interfere by establishing a rate-in-aid, much to the discredit of the wealthiest city in the world. The hospitals must be maintained in a thoroughly efficient condition, come what may, and if the metropolitan community lacks the public spirit to voluntarily subscribe the requisite funds, compulsion will have to be applied.

**Pall Mall Gazette** for December 26th, 1891.

What is the remedy for that serious falling-off in the subscriptions to the London hospitals to which we drew attention the other day? That is a question not inappropriate to the present season of charity and of charitable appeals. One possible cause we have already discussed. The discussion which has waged during the year in newspapers and magazines over a series of "Hospital Scandals"—whether rightly or wrongly so called—has suggested doubts as well as quickened interest. Some of the scandals have been in public and non-voluntary institutions, but the public is not given to careful discrimination. These are things which, when charitable persons are fingering their purse-strings, *donnent furieusement à penser*. We have no doubt that many such are keeping back their subscriptions until they feel satisfied that there is not "something wrong." It is a natural, if a lazy and selfish, way out of their perplexity. But while they ponder the poor die. The first to suffer by a diminution of funds are not the officials of the hospitals, but the sick and helpless working folk for whom the hospitals exist. What, then, is to be done?

The first thing, of course, is for the authorities to leave off wringing their hands over the "agitation" and set to work to remove its cause. This is not to be done by addressing to the "agitators" alternate insults and appeals, and to the public vague assurances that all is for the best in the best of all possible philanthropies. In enormous institutions like these, coping with a mass of difficult work, there must arise mistakes and injustices and defects. Let the hospital authorities frankly recognise this; let them, instead of meeting publicity with an angry howl about endangering subscriptions, welcome it and turn it to advantage; let them say, if it be true, that they cannot undertake to be just to nurses as well as kind to patients unless they have more money; and they will soon find that the money is forthcoming. . . . The great voluntary hospitals are in theory governed by a democracy of the whole body of subscribers. In practice, of course, most of them are governed by small knots of well-meaning old gentlemen, who have a certain amount of time on their hands and a *penchant* for the fashionable character of philanthropist. Among them are soft hearts (and heads) against whom we should be sorry to say a word. But, taken as a class, of the details of the administration over which they preside they are profoundly ignorant; they hate having to get things up, or take a side, or do anything more than form an occasional quorum, and at rarer intervals take a walk through a ward under the safe conduct of one of the paid officials, whom they would as soon fly as contradict. What is needed in the committees of these worthies is the infusion of a few energetic and business-like women, with leisure enough to devote to the work and with the practical feminine eye for detail. But meanwhile something might be done with the Quarterly Courts at which the committee has to submit its minutes. For most subscribers these Courts are held at a most inconvenient place at the most inconvenient hour. But we are assuming the willingness to take a certain amount of trouble for a season or so. Let them attend these Courts, attend to any points of criticism raised by the more ardent reformers, and insist on having such points either fairly met or frankly conceded. The formula, in other words, for such subscribers as decline to sacrifice one set of human beings for the alleged benefit of another is this: Do not give less of your money, but give more of yourself.

**The Star** for December 24th, 1891.

The editor of The Hospital and other publications in the interests of private charity, issues a Christmas appeal on behalf of the metropolitan hospitals. The hospitals have experienced a period of depression during the present year, and according to the writer, unless contributions roll in during the next few days there will be a deficiency of £150,000. We hope the wealthy philanthropists will attend to this and be liberal with their Christmas contributions. If Mr. Burdett, who acts as a sort of Father Christmas for the hospitals, had contented himself with a simple appeal, further remark would be unnecessary; but he goes out of his way to laud the voluntary hospitals and decry rate-supported institutions. Here we must join issue with him. The hospitals in London are far from models of careful administrations. Ugly scandals connected with their management are continually coming to light. For mismanagement and corruption we would couple such voluntary establishments as St. Bartholomew's and the London Hospitals, the Homerton Hospital, which is supported by the rates, but is unfortunately controlled by a body which is not directly elected by the ratepayers, and is not responsible to them. The only rate-supported institutions in the metropolis which are directly managed by the people's representatives are the lunatic asylums under the County Council, and he cannot point to a voluntary hospital in London which is half so well managed in the interests of patients and public alike as are these institutions.

**Echo for Dec. 19th, 1891.**

Everybody knows that in the provincial towns a much larger sum per thousand of the population is subscribed for the support of local hospitals and charities than is the case in London. There is, however, a set of statistics in "Burdett's Hospital Annual" for 1891-92 which will surprise people who have not hitherto taken the trouble tc look into the figures. Statistics show that the artizan classes in the principal provincial towns contribute to their hospitals nearly £60,000 a year at a cost of £2,410, or less than 5 per cent., as against £16,000 subscribed in London at a cost of £2,961, or more than 18 per cent. Our hospitals are still in a bad way, and unhappily their prospects are not improving. It is anticipated that the deficit on the operations of the present year, instead of being £84,000, as in 1890, will be not less than £150,000.

**The Guardian for December 23rd, 1891.**

It is bad news just at the beginning of the real winter that, during 1891, the income of the London hospitals has fallen off by £150,000. We cannot say, however, that it surprises us. There is nothing which people seem slower to learn than that the charity fund is pretty well fixed, and that the differences which it shows from time to time relate less to amount than to distribution. When General Booth got his £100,000, careful observers predicted that it would be mainly drawn from the subscription lists of other charities, and what is true of this fund is true of all other new charitable undertakings. They live on money diverted from older undertakings of the same kind. We do not say, of course, that no new charitable undertakings ought to be supported. But we do say that they ought not to be supported without full consideration of the effect which they are calculated to have on those already in existence. Everyone who is applied to on behalf of a new charity ought to ask himself first whether the place is not already filled—in which case he will do better to increase the income of the older institution, and so save in cost of management—and next, whether, even if the place be not already filled, it is worth while filling it at the cost of the inevitable loss which will be caused to kindred though not identical institutions. What is wanted is not more charities, but more charity, not new ways in which to spend money, but a more liberal expenditure in the old ways. If this warning had been borne in mind during the last twelve months we should not now need to be reminded that the hospitals of London alone are £150,000 to the bad.

**The National Observer for December 26th, 1891.**

A certain fixed sum, economists tell us, is annually spent upon charity. If that sum be drawn off by adroit advertisement into unprofitable channels, deserving enterprises suffer. The past year has witnessed the triumph of *blague*; and while we have no sympathy for the victims who poured their thousands into "General" Booth's hat, we grievously deplore the deficit in the hospital funds which is indeed an indirect consequence of the Booth subscription. Concerning the admirable work performed by our hospitals there is and can be no question. They at least rest on sounder foundations than vulgar advertisement and religious hysteria. And yet, because the people's ear is irritated by the shrieks of the fanatic, the usefulness of the best institutions in the world must needs be imperilled. The past year has been the worst that the London hospitals have ever encountered, and we commend this fact to the consideration of those modern philanthropists whose right hand declines to give unless the generosity be proclaimed to their left by the beating of drums and the tinkling of cymbals. Let him who is in doubt how to bestow his charity remember that wise work efficiently accomplished is better worth than the noisiest advertisements of "blood and fire." And if only common sense again prevail it may be not ours next year to record that while "General" Booth has spent £100,000, and still clamours for more, the hospitals are burdened with a deficit of £150,000.

**Church Times for December 26th, 1891.**

The income of the hospitals has seriously fallen off, not only from the depression which has affected those fortunate enough to be endowed, but also from shrinkage of income from subscriptions and legacies. People probably now contribute to the hospital fund instead of sending a subscription direct to headquarters. That would be well and good, if it were not that in many cases where a guinea was formerly sent up as a subscription, half-a-guinea, if so much, is now placed in the alms bag in church. This is a form of "hedging" which may appeal to the humorous side of a member of the betting fraternity, but calls for a sterner description from every honest man. To look facts in the face, we understand that the hospitals will probably have to face a deficiency of £150,000, owing to the shrinkage which has taken place in their incomes, and seeing the enormous value of these institutions to the great bulk of the population, not lessened by some palpable but probably unavoidable abuses, it is a matter which should appeal to the public conscience to see that those who have not hitherto helped to support the hospitals should do so. Is the piteous tale poured forth by hospital treasurers another result of charity diverted into the untried channel opened by "General" Booth? It looks like it.

**The Graphic for December 26th, 1891.**

It is anything but glad tidings at this season of the year to learn that the London hospitals will be about £150,000 to the bad on New Year's Day, unless some unexpected and very substantial windfall is at once forthcoming. A writer, who gives this depressing information in the current number of THE HOSPITAL, states that it rests on "the most unquestionable authority." It may be safely assumed, therefore, that somewhere about that sum will have to be made good from outside sources during next year, if the hospitals are to be saved from closing some more of their wards. Last year, at this time, the deficit only amounted to £84,173, and about half was wiped off by the Saturday and Sunday collections. But, although those excellent funds grow a little every year, it would be futile to anticipate such a huge expansion as would be required to even half cover the present gigantic deficit. The writer, therefore, calls upon the Press to give whatever help it can in stimulating public generosity, and to this praiseworthy appeal we respond with all possible good will. But we are not very sanguine as to the result; the financial world, which is always a most liberal supporter of deserving charities, has suffered very heavy losses, nor is trade in a sufficiently flourishing condition to justify expectation of increased help from that quarter. The likelihood seems to be, therefore, that 1892 will prove very similar to 1891 in regard to the exceptional difficulty of "procuring money for hospitals." What is to be done, then? That is becoming a graver and more pressing question every year. That the London hospitals should be, and must be, maintained in thorough efficiency will be disputed by no one. But maintenance is a question of money, and if that be not forthcoming to the required extent through voluntary instrumentality, there will be nothing for it but to apply compulsion in the form of a hospital rate-in-aid.

**The Gentlewoman for December 26th, 1891.**

CHRISTMAS CHARITY.

Our duties! Our charities! "Charity," some of you cynics may say, "covers a multitude of sins." Well, be it so! But charity covereth the naked, the miserable, and the suffering—there is, as Carlyle would have had it, "a real fact in charity." It *does* do good. This day I have had a letter written to me, aye, two or three or four or five letters to poor Mrs. Gentlewoman, saying that times are bad and things are sad. The hospitals are *not* doing well. Ye gentlewomen of England, who live at home at ease, just for a few minutes think over this. "I pray you, in your charity." You are happy! Well, if you are cynical, say, more or less so. I am sure that you are, more or less. So think of those hospitals where funds are not forthcoming for beds, and gear, and hospital wear. Do not be less generous to your friends—but be a little self-sacrificing to yourselves. Help those who want help. Those who want help are the poor and the hungry and the hospitals. "I pray you, in your charity," be kind to all those about you. There are Christmas duties, as well as Christmas pleasures. And please bear this in mind. How sad those appeals of the hospitals. "I regret to say," &c.—" depression," &c.—" we want." And, in Christmas spirit, really you, my friends, should do your best to supply those wants. Let it be a happy season for one and all.

